# Comparison of Osteogenic Differentiation Potential of Human Dental-Derived Stem Cells Isolated from Dental Pulp, Periodontal Ligament, Dental Follicle, and Alveolar Bone

**DOI:** 10.1155/2021/6631905

**Published:** 2021-04-07

**Authors:** Guanlin Qu, Yan Li, Lu Chen, Qin Chen, Duohong Zou, Chi Yang, Qing Zhou

**Affiliations:** ^1^Department of Oral and Maxillofacial Surgery, School and Hospital of Stomatology, China Medical University, Liaoning Provincial Key Laboratory of Oral Diseases, Shenyang, Liaoning, China; ^2^Department of Oral Surgery, Shanghai Ninth People's Hospital, Shanghai Jiao Tong University School of Medicine; College of Stomatology, Shanghai Jiao Tong University; National Center for Stomatology; National Clinical Research Center for Oral Diseases; Shanghai Key Laboratory of Stomatology; Research Unit of Oral and Maxillofacial Regenerative Medicine, Chinese Academy of Medical Sciences, Shanghai, China

## Abstract

**Background:**

Mesenchymal stem cells (MSCs) have become promising candidates for regeneration medicine due to their multidifferentiation potential and immunomodulatory ability. Compared with classic MSCs derived from the bone marrow and fat, dental-derived MSCs show high plasticity, accessibility, and applicability. Therefore, they are considered alternative sources for regeneration medicine.

**Methods:**

Four types of MSCs were isolated from the dental pulp, periodontal ligament, dental follicle, and alveolar bone of the same donor, and there were five different individuals. We analyzed their morphology, immunophenotype, proliferation rate, apoptosis, trilineage differentiation potential, and the gene expression during osteogenic differentiation.

**Results:**

Our research demonstrated that DPSCs, PDLSCs, DFPCs and ABMMSCs exhibited similar morphology and immunophenotype. DFPCs showed a higher rate of proliferation and apoptosis. When cultured in the trilineage differentiation medium, all types of MSCs presented the differentiation potential of osteogenesis, adipogenesis, and chondrogenesis. Through staining and genetic analysis during osteogenic induction, ABMMSCs and PDLSCs showed the highest osteogenic ability, followed by DPSCs, and DFPCs were the lowest.

**Conclusions:**

Overall, our results indicated that different dental-derived stem cells possessed different biological characteristics. For bone tissue engineering, ABMMSCs and PDLSCs can be used as optimal candidates of seed cells.

## 1. Introduction

Bone regeneration is a challenging issue in clinical treatment. A variety of causes such as trauma, congenital abnormalities, or tumor resection, can destroy the bone and cause dysfunction. To date, autologous tissue transplantation provides a possible treatment method with the development of functional reconstruction surgery [1]. However, due to the shortcomings such as insufficient donor tissue sources and wound infection, functional reconstruction is limited [2]. In recent years, bone tissue engineering based on stem cells, biomaterials, and bioactive macromolecules is becoming a new field in the reconstruction of bone defect and the selection of seed cells is a crucial element [3]. The ideal seed cells should possess the advantages including a wide range of sources, easy extraction, rapid expansion, and multilineage differentiation potential. In addition, stem cells should show the ability of combining with scaffold materials stably and forming a fitting microenvironment [4].

Mesenchymal stem cells (MSCs) are pluripotent progenitor cells that can differentiate into different kinds of lineages, including osteoblasts, adipocytes, chondrocytes, epithelial cells, neuron-like cells, and hepatocyte-like cells [5]. These characteristics make MSCs be attractive treatments for regenerative medicine, especially the repair and reconstruction of bone defect. In addition, due to the low immunogenicity and significant immunomodulatory capacity, MSCs are considered ideal candidates for therapeutic applications. After the first successful isolation from the bone marrow, MSCs were subsequently isolated from various other tissues, such as the adipose tissue, umbilical cord, amnion, teeth, and synovium [6–10]. In recent years, dental-derived MSCs have drawn much attention owing to their plasticity, accessibility, and applicability. However, the ideal dental-derived MSCs for tissue regeneration remain to be elucidated.

So far, a variety of MSCs from dental tissues have been isolated and characterized, including dental pulp stem cells (DPSCs) [11], periodontal ligament stem cells (PDLSCs) [12], dental follicle progenitor cells (DFPCs) [13], alveolar bone-derived MSCs (ABMMSCs) [14], stem cells from human shedding deciduous teeth (SHED) [15], apical papillary stem cells (SCAP) [16], tooth germ progenitor cells (TGPCs) [17], and gingival MSCs (GMSCs) [18]. Because bone marrow mesenchymal stem cells (BMMSCs) have been widely used in clinic treatment, the characteristics of dental-derived MSCs are usually compared with BMMSCs. Similar to BMMSCs, dental-derived MSCs can differentiate into three types of cell lineages at least, and they also exhibit different characteristics. Moreover, dental-derived MSCs show the advantages of immunomodulation and anti-inflammatory properties in the local environment [19]. However, no studies have systematically compared the characteristics of DPSCs, PDLSCs, DFPCs, and ABMMSCs which can be easily obtained from the extracted third molars and their surrounding tissues.

In this study, we replaced traditional BMMSCs with ABMMSCs that can be obtained with low trauma and systematically compared the morphology, immunophenotype, proliferation rate, antiapoptosis ability, and trilineage differentiation potential of DPSCs, PDLSCs, DFPCs, and ABMMSCs. The osteogenic ability and gene expression patterns in the osteogenic process of four types of stem cells were compared to evaluate their clinical application potential in repairing bone defect in order to provide theoretical support for the selection of the best seed cells of bone tissue engineering.

## 2. Materials and Methods

### 2.1. Cell Isolation and Culture

The healthy teeth and alveolar bone samples came from the extracted third molar, and the donors were 16-20 years old. Each type of MSC coming from the same donor was used for the following experiments, and there were five different individuals. All the procedures were approved by the Research Ethics Committee of Shanghai Jiao Tong University School of Medicine (SH9H-2020-TK60-1). For the isolation of DPSCs, PDLSCs, and DFPCs, the dental pulp tissue, dental follicle tissue, and one-third of the periodontal ligament tissue on the root surface were washed with phosphate-buffered saline (PBS) and cut into small pieces of 1-2 mm^3^. Then, they were digested with 3 mg/mL type I collagenase (Sigma, USA) for 40 minutes at 37°C. After that, the tissues were passed through a 70 *μ*m strainer (Thermo Scientific, USA); then, the suspension was centrifuged at 1200 rpm for 5 minutes. The cell pellets were resuspended in MEM-*α* medium (Biolnd, Israel) containing 20% fetal bovine serum (FBS, Biolnd, Israel) and 100 U/mL of penicillin-streptomycin (Beyotime, China) and then were plated into dishes. For the isolation of ABMMSCs, the alveolar bone was crushed into small pieces and seeded in dishes using the same medium. All the types of cells were incubated at 37°C in a humidified atmosphere with 5% CO_2_. At around 80% confluence, cells were detached by 0.25% trypsin (Beyotime, China) and split at the ratio of 1 : 3. The medium for all types of cells was refreshed every 3 days. Cells at passage 3 were utilized for further experiments.

### 2.2. Flow Cytometry

The four types of MSCs at passage 3 were harvested by trypin-EDTA (Sigma, USA) and centrifuged at 1200 rpm for 5 minutes. Then, the cell pellets were resuspended in PBS and transferred to the test pipe. The immunophenotype was analyzed with the following antibodies: FITC-conjugated CD24, CD29, CD44, CD45, and CD73 and PE-conjugated CD34, CD90, CD105, and CD146. Corresponding isotype-matched antibodies were used as controls. All the antibodies were purchased from BD Bioscience (NJ, USA). Cells were incubated in the dark for 30 minutes and collected into a flow cytometer. The results were analyzed by using Cell-Quest for Macintosh Software.

### 2.3. TUNEL Staining

MSCs were cultured on cell slides until reaching 80% confluence and then replaced with serum-free medium for 24 hours. Then, they were fixed with 4% PFA for 30 minutes and washed 3 times with PBS. Then, we followed the manufacturer's instructions of TUNEL detection kit (Servicebio, China) and took pictures under a fluorescence microscope. The red light represented the apoptotic cell nucleus stained by TUNEL, and the blue light was the cells stained by DAPI. The percentage of the number of red fluorescent cells to the number of blue fluorescent cells represented the proportion of apoptotic cells in the total cells.

### 2.4. Cell Proliferation

According to the manufacturer's instructions, Cell Counting Kit-8 (Dojindo, Japan) was used to detect cell proliferation. The four types of MSCs were seeded in 96-well plates (Corning, USA) at a density of 3 × 10^3^ cells/well, and each well contained 100 *μ*L *α*-MEM with 10% FBS. After the incubation for the first 24 h, the cell number was tested every 24 h for seven consecutive days. To determine the number of MSCs, 10 *μ*L of CCK-8 reagent was added to each well and then incubated for 2 hours at 37°C. The optical density value at a wavelength of 450 nm was detected with Multiskan GO (Thermo Scientific, USA). Each experiment was performed in three replicates.

### 2.5. Adipogenic Differentiation

For adipogenic differentiation, the four types of MSCs were seeded in each well of 6-well plates at the density of 2 × 10^5^ cells/well and maintained in MEM-*α* medium containing 10% FBS. When cells reached 100% confluency, the medium was replaced with the adipogenic differentiation medium (Cyagen, USA) according to the manufacturer's instructions and kept for 21 days. Lipid droplets of the resultant differentiated cells were detected using Oil red staining (Cyagen, USA).

### 2.6. Chondrogenic Differentiation

For chondrogenic differentiation, 5 × 10^5^ cells were centrifuged in 15-mL polypropylene tube (Corning, USA) and resuspended in 500 *μ*L chondrogenic differentiation medium (Cyagen, USA). The medium was refreshed every 3 days. After 21 days, the cell pellets were fixed in 4% PFA and then cut into paraffin sections of 5 *μ*m thickness. To assess the deposition of glycosaminoglycans, the sections were stained with alcian blue (Cyagen, USA).

### 2.7. Osteogenic Differentiation

For osteogenic differentiation, the MSCs were seeded in each well of 6-well plates at the density of 1 × 10^5^ cells/well and maintained in MEM-*α* medium containing 10% FBS. When the cells reached 60-70% confluence, the medium was replaced with the osteogenic differentiation medium (Cyagen, USA). Cells were then seeded in osteogenic differentiation medium for 21 days, and the medium was refreshed every 3 days.

### 2.8. ALP Activity Staining and Assay

After osteogenic differentiation, the MSCs were detected by ALP staining kit (Jiancheng Biotech, China) and ALP activity assay kit (Solarbio, China) at days 3, 7, and 14. The cells were incubated in ALP staining solution for 20 minutes at 37°C. A scanner (HP, China) was used to capture digital images. ALP activity was normalized by the protein concentration. Following standard protocols, the proteins were extracted using RIPA lysis buffer (Sigma, USA) and concentrations were measured by Bradford protein assay kit (Sangon Biotech, China).

### 2.9. Alizarin Red Staining and Calcium Deposit Quantification

After osteogenic differentiation for 21 days, the MSCs were detected for mineralized nodules by using alizarin red staining solution (Cyagen, USA). The cells were fixed in 4% PFA for 30 minutes and incubated with alizarin red staining solution at room temperature. After 5 minutes, the reactions were then stopped by washing with PBS. A scanner and microscope was used to capture digital images. After staining with alizarin red, the calcium nodules were dissolved by 10% CPC and incubated at 37°C for 30 minutes. Then, the supernatant was transferred to a 96-well plate, and the absorbance at 590 nm was measured for statistical analysis.

### 2.10. RNA Extraction and qRT-PCR

Total RNA was extracted by using RNAiso Plus (Takara, Japan) during trilineage differentiation of the MSCs. RNA concentration and purity were measured by using a Multiskan GO (Thermo Scientific, USA). The cDNA was synthesized from RNA by using a retrotranscription kit (Takara, Japan). Quantitative real-time polymerase chain reaction (qRT-PCR) was performed using SYBR Green I master mix (Takara, Japan) and running in a LightCycler 96 (Roche, CH). Primer sequences are outlined in Table 1. The relative expression of each gene was performed using 2^-*ΔΔ*Ct^ values obtained by normalization to *β*-actin RNA as the internal control.

### 2.11. Western Blotting

After 21 days of osteogenic differentiation of DPSCs, PDLSCs, DFPCs, and ABMMSCs, the total cellular protein was extracted using the RIPA lysis buffer (Sigma, USA), and concentrations were measured by Bradford protein assay kit (Sangon Biotech, China). MSCs that were cultured in MEM-*α* medium containing 10% FBS were used as a control. The samples were heated for 8 min at 100°C. Equal amount of proteins was separated by 10% SDS-polyacrylamide gel and transferred to polyvinylidene difluoride (PVDF) membranes (Bio-Rad, USA). The membranes were blocked with 5% skim milk for 2 hours at room temperature and then incubated with primary antibodies RUNX2 (1 : 2000, ab236639, Abcam), ALP (1 : 2000, ab83259, Abcam), OSX (1 : 2000, ab94744, Abcam), OPN (1 : 2000, ab8448, Abcam), and *β*-actin (1 : 2000, ab8227, Abcam) overnight at 4°C. After washing with TBST buffer, the blots were incubated with HRP goat anti-rabbit (1 : 1000, Abcam) for 1 hour at room temperature. The proteins were visualized using a ChemiDoc imaging system (Bio-Rad, USA).

### 2.12. Statistical Analysis

All statistical analyses were calculated using GraphPad Prism 8 Software. Differences among more than three groups, one-way ANOVA was applied. All experiments were repeated three times at least, and the data are presented as mean ± SD. Results were considered statistically significant with *p* value; *p* < 0.05 was considered statistically significant (^∗^*p* < 0.05, ^∗∗^*p* < 0.01, and ^∗∗∗^*p* < 0.001 vs. DPSCs; ^#^*p* < 0.05, ^##^*p* < 0.01, and ^###^*p* < 0.001 vs. PDLSCs; ^&^*p* < 0.05, ^&&^*p* < 0.01, and ^&&&^*p* < 0.001 vs. DFPCs; ^†^*p* < 0.05, ^††^*p* < 0.01, and ^†††^*p* < 0.001 vs. ABMMSCs).

## 3. Results

### 3.1. MSC Morphology

**Figure 1:**
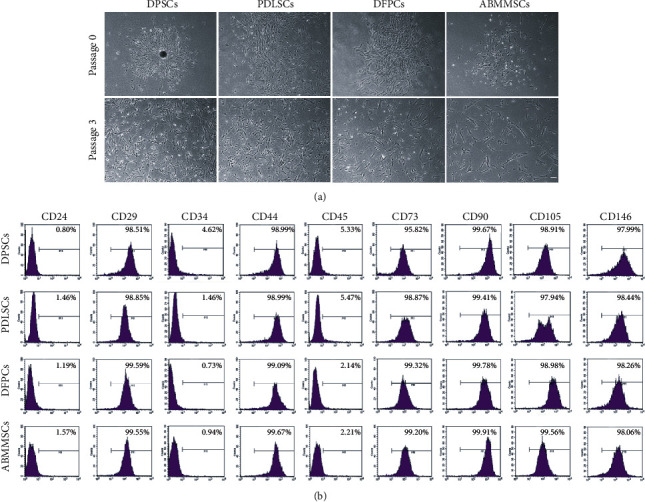
Morphology and immunophenotype of DPSCs, PDLSCs, DFPCs, and ABMMSCs. (a) Phase-contrast microscope images of MSCs at passage 0 and passage 3 (scale bar = 1 mm, magnification = ×40). (b) Representative pictures of flow cytometric analysis of the surface marker expression on DPSCs, PDLSCs, DFSCs, and ABMMSCs.

After isolating and cultivating DPSCs, PDLSCs, DFPCs and ABMMSCs, the MSCs were expanded and passaged in the culture medium. An inverted phase-contrast microscope was used to evaluate the morphology of the MSCs at passages 0 and 3 (Figure 1(a)). We found that all types of MSCs exhibited a fibroblast-like long spindle shape and showed a swirling arrangement when converging. After 3 passages, the cell morphology was still smooth and consistent. No significant difference in morphology was observed among the four types of cells.

### 3.2. MSC Immunophenotype

We analyzed the immunophenotype of MSCs by flow cytometry. The data showed a low expression of endothelial and hematopoietic markers (CD24, CD34, and CD45) and high expression of typical MSC markers (CD73, CD90, and CD105) and CD29, CD44, and CD146 (Figure 1(b)). The results showed that there was no difference among the four types of MSCs in terms of immunophenotype. It was reported that CD146-positive MSCs have higher proliferation and differentiation ability than CD146-negative MSCs [20]. In order to eliminate the interference of CD146, we selected the MSCs which expressed CD146 highly (>95%) for the following tests.

### 3.3. Apoptosis and Proliferation Rates

**Figure 2:**
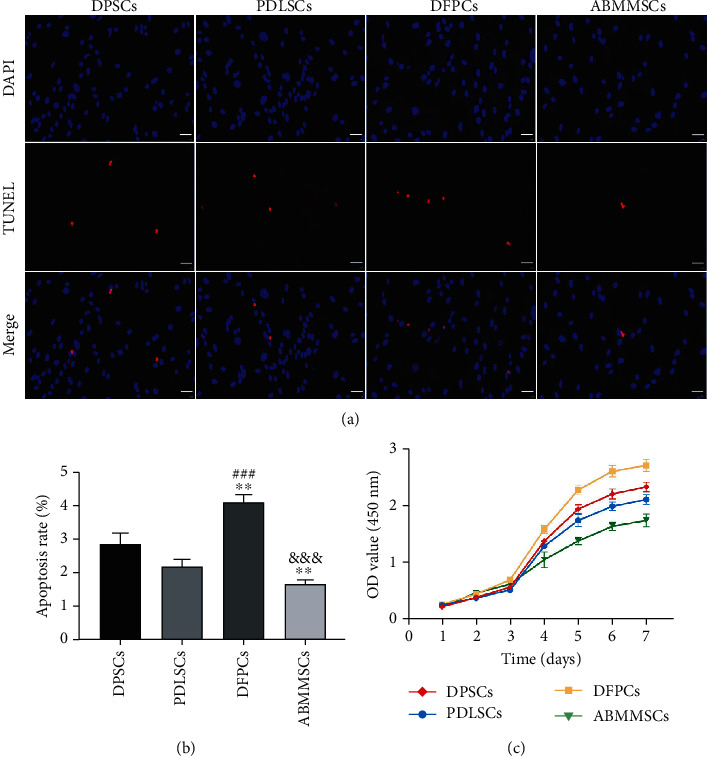
Apoptosis and proliferation rates of DPSCs, PDLSCs, DFPCs, and ABMMSCs. (a) TUNEL staining of DPSCs, PDLSCs, DFPCs, and ABMMSCs at P3 (scale bar = 50 *μ*m). (b) The apoptosis rate of DPSCs, PDLSCs, DFPCs, and ABMMSCs at P3. (c) The proliferation rate of DPSCs, PDLSCs, DFPCs, and ABMMSCs at P3 (^∗^*p* < 0.05, ^∗∗^*p* < 0.01, and ^∗∗∗^*p* < 0.001 vs. DPSCs; ^#^*p* < 0.05, ^##^*p* < 0.01, and ^###^*p* < 0.001 vs. PDLSCs; ^&^*p* < 0.05, ^&&^*p* < 0.01, and ^&&&^*p* < 0.001 vs. DFPCs; ^†^*p* < 0.05, ^††^*p* < 0.01, and ^†††^*p* <0.001 vs. ABMMSCs).

We induced the MSC apoptosis through serum deprivation. The results showed that the apoptotic rate of DFPCs was the highest and that of ABMMSCs was the lowest, followed by DPSCs and PDLSCs. There was no statistical difference in the apoptotic rate between DPSCs and PDLSCs (Figures 2(a) and 2(b)).

According to the CCK-8 test results for consecutive 7 days, the growth curve was analyzed and drawn. The results showed that all the MSCs proliferated slowly in the first two days and entered the logarithmic phase at the third day. The proliferation rate of DFPCs was significantly faster than that of ABMMSCs, and DPSCs and PDLSCs were the second (Figure 2(c)).

### 3.4. ALP Activity Staining and Assay

**Figure 3:**
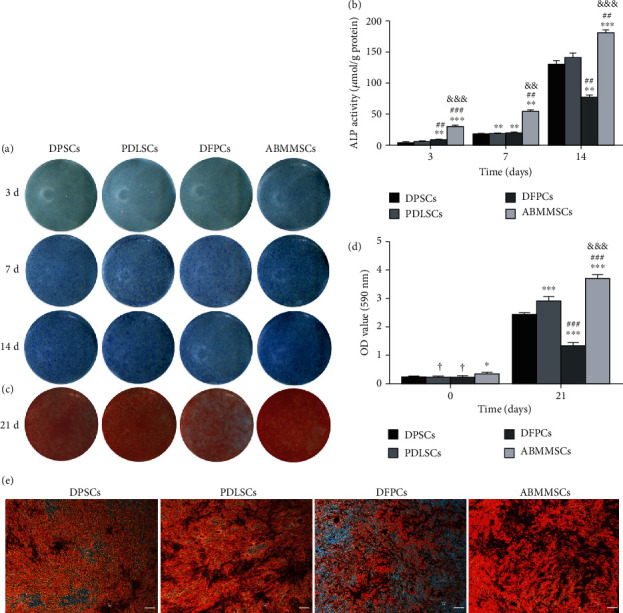
Osteogenic differentiation staining of DPSCs, PDLSCs, DFPCs, and ABMMSCs. (a) ALP staining of DPSCs, PDLSCs, DFPCs, and ABMMSCs after 3, 7, and 14 days of osteogenic differentiation (*n* = 3). (b) ALP activity assay of DPSCs, PDLSCs, DFPCs, and ABMMSCs after 3, 7, and 14 days of osteogenic differentiation (*n* = 3). (c) Alizarin red staining of DPSCs, PDLSCs, DFPCs, and ABMMSCs after 21 days of osteogenic differentiation (*n* = 3). (d) Alizarin red staining for analysis of the calcium deposition amount of the DPSCs, PDLSCs, DFPCs, and ABMMSCs after 21 days of osteogenic differentiation. (*n* = 3). (e) Alizarin red staining of DPSCs, PDLSCs, DFPCs, and ABMMSCs after 21 days of osteogenic differentiation (*n* = 3) (scale bar = 2 mm) (^∗^*p* < 0.05, ^∗∗^*p* < 0.01, and ^∗∗∗^*p* < 0.001 vs. DPSCs; ^#^*p* < 0.05, ^##^*p* < 0.01, and ^###^*p* < 0.001 vs. PDLSCs; ^&^*p* < 0.05, ^&&^*p* < 0.01, and ^&&&^*p* < 0.001 vs. DFPCs; ^†^*p* < 0.05, ^††^*p* < 0.01, and ^†††^*p* < 0.001 vs. ABMMSCs).

ALP is mainly expressed in the mineralized matrix produced by osteogenic differentiated cells and is considered to be an early marker of osteoblast [21]. In the process of osteogenic induction, the ALP activity of the four types of MSCs increased with time. At days 3, 7, and 14 of osteogenic induction, ABMMSCs showed the highest ALP activity, followed by PDLSCs and DPSCs, and DPFCs possessed the lowest (Figures 3(a) and 3(b)).

### 3.5. Alizarin Red Staining and Calcium Deposit Quantification

In order to detect the osteogenic capacity of DPSCs, PDLSCs, DFPCs, and ABMMSCs, we performed osteogenic differentiation experiments. Each type of cells was cultured in osteogenic differentiation medium for 21 days. The staining results confirmed that all the MSCs had undergone osteogenic differentiation, but the efficiency was different. As shown in Figures 3(c)–3(e), ABMMSCs and PDLSCs showed stronger alizarin red staining than DFPCs, followed by DPSCs, which indicated that the osteogenic capacity declined from ABMMSCs, PDLSCs, and DPSCs to DFPCs. In order to quantify the calcium deposition level of the cell substrates, the deposited calcium was dissolved in 10% CPC and quantified using a microplate reader (Figure 3(d)). As expected, the calcium levels in ABMMSCs and PDLSCs were higher compared to DPSCs and DFPCs.

### 3.6. Osteogenic Gene Expression Analysis

**Figure 4:**
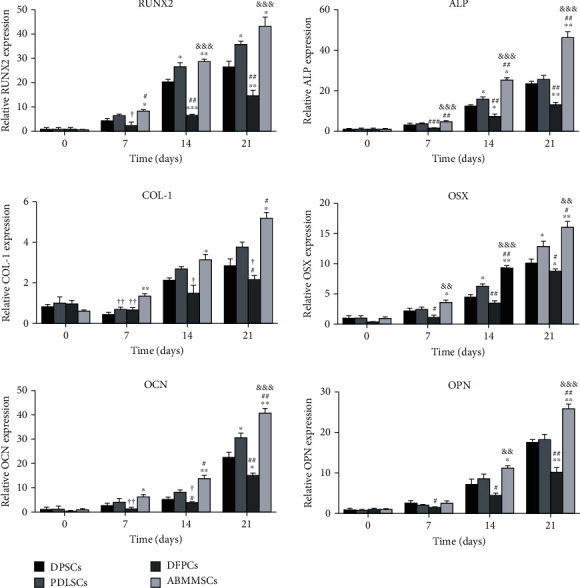
Gene expression for osteogenic differentiation. qRT-PCR was performed to analyze the potency of osteogenic differentiation in DPSCs, PDLSCs, DFPCs, and ABMMSCs at days 0, 7, 14, and 21. The *y*-axis represents the relative mRNA fold change, which was calculated using the 2^−*ΔΔ*Ct^ formula with *β*-actin as the internal control. Error bars represent the SD (*n* = 3) (^∗^*p* < 0.05, ^∗∗^*p* < 0.01, and ^∗∗∗^*p* < 0.001 vs. DPSCs; ^#^*p* < 0.05, ^##^*p* < 0.01, and ^###^*p* < 0.001 vs. PDLSCs; ^&^*p* < 0.05, ^&&^*p* < 0.01, and ^&&&^*p* < 0.001 vs. DFPCs; ^†^*p* < 0.05, ^††^*p* < 0.01, and ^†††^*p* < 0.001 vs. ABMMSCs).

We further evaluated the osteogenic capacity of MSCs by comparing the relative mRNA expression of osteogenic markers at days 0, 7, 14, and 21 of osteogenic induction (Figure 4). Except for COL-1 in DPSCs, PDLSCs, and DFPCs which showed a downward trend at day 7 of osteogenesis, OPN, OCN, RUNX2, OSX, and ALP in the MSCs all increased with time. At the 21st day of osteogenic induction, the results showed that all the markers were expressed highest in ABMMSCs, lowest in DFPCs, and DPSCs and PDLSCs were the second.

**Figure 5:**
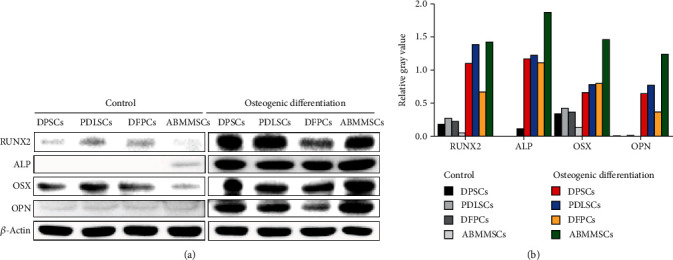
Protein expression for osteogenic differentiation. (a) Western blot analysis of osteogenic markers (RUNX2, ALP, OSX, and OPN) proteins in DPSCs, PDLSCs, DFPCs, and ABMMSCs with and without 21 days of osteogenic differentiation (*n* = 3). (b) The relative gray value of RUNX2, ALP, OSX, and OPN/*β*-actin.

We also examined the expression of osteogenic differentiation protein markers RUNX2, ALP, OSX, and OPN using western blotting (Figures 5(a) and 5(b)). After 21 days of osteogenic induction, OSX and ALP were expressed at higher levels in all the MSCs compared with the control group. In addition, the osteogenic transcription factor RUNX2 and the late marker OPN were significantly enhanced in ABMMSCs, PDLSCs, and DPSCs, but only showed slightly higher protein levels in DFPCs than the control cells. In summary, these findings indicate that MSCs isolated from different dental-derived tissues exhibit unique osteogenic differentiation capacity, and there are significant differences in the expression of osteogenesis-related genes among them. Taken together with the alizarin red staining results, it was concluded that among the four types of MSCs, the osteogenic efficiency was highest in ABMMSCs, followed by PDLSCs, moderate in DPSCs, and lowest in DFPCs.

### 3.7. Adipogenic Differentiation and Gene Expression Analysis

**Figure 6:**
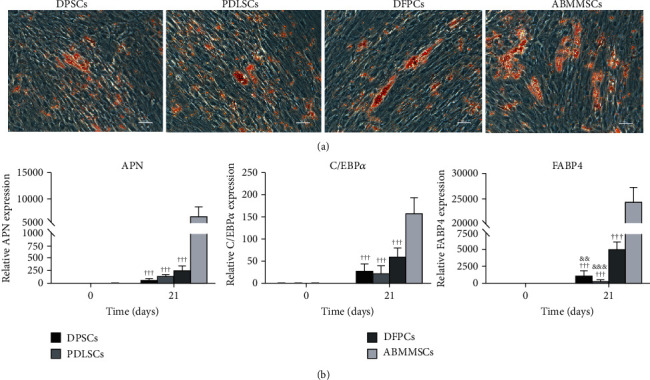
Adipogenic differentiation capacity of DPSCs, PDLSCs, DFPCs, and ABMMSCs. (a) Oil red O staining of DPSCs, PDLSCs, DFPCs, and ABMMSCs after 21 days of adipogenic differentiation (scale bar = 500 *μ*m). (b) qRT-PCR was performed to analyze the potency of adipogenic differentiation in DPSCs, PDLSCs, DFPCs, and ABMMSCs at days 0 and 21. The *y*-axis represents the relative mRNA fold change, which was calculated using the 2^−*ΔΔ*Ct^ formula with *β*-actin as the internal control. Error bars represent the SD (*n* = 3) (^∗^*p* < 0.05, ^∗∗^*p* < 0.01, and ^∗∗∗^*p* < 0.001 vs. DPSCs; ^#^*p* < 0.05, ^##^*p* < 0.01, and ^###^*p* < 0.001 vs. PDLSCs; ^&^*p* < 0.05; ^&&^*p* < 0.01, and ^&&&^*p* < 0.001 vs. DFPCs; ^†^*p* < 0.05, ^††^*p* < 0.01, and ^†††^*p* < 0.001 vs. ABMMSCs).

In order to test the adipogenic capacity of DPSCs, PDLSC, DFPCs, and ABMMSCs, the MSCs were cultured in the adipogenic induction medium. At about 7 days after induction, the MSCs became flat and lipid vacuoles began to appear in the induced cells. After 21 days of induction, the cells were fixed by 4% PFA and then the adipogenesis was verified by oil red O staining. The accumulation of cytoplasmic oil droplets could be clearly observed in the four types of MSCs (Figure 6(a)). We further evaluated the adipogenic capacity of MSCs by comparing the relative mRNA expression of adipogenic markers at days 0 and 21 of adipogenic induction (Figure 6(b)). The results showed that all the adipogenic markers were expressed highest in ABMMSCs.

### 3.8. Chondrogenic Differentiation and Gene Expression Analysis

**Figure 7:**
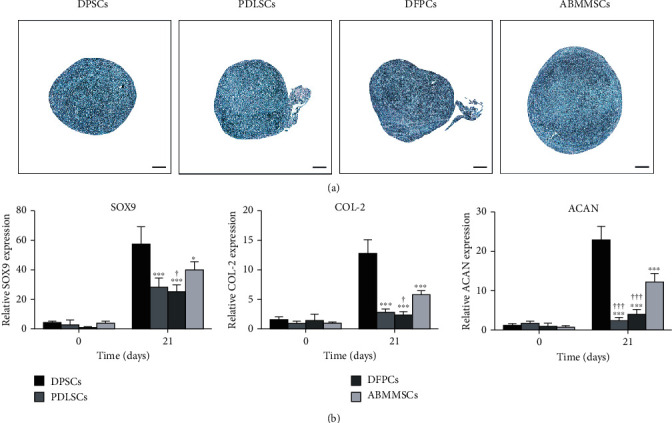
Chondrogenic differentiation capacity of DPSCs, PDLSCs, DFPCs, and ABMMSCs. (a) Alcian blue staining of DPSCs, PDLSCs, DFPCs, and ABMMSCs after 21 days of chondrogenic differentiation (scale bar = 200 *μ*m). (b) qRT-PCR was performed to analyze the potency of chondrogenic differentiation in DPSCs, PDLSCs, DFPCs, and ABMMSCs at days 0 and 21. The *y*-axis represents the relative mRNA fold change, which was calculated using the 2^−*ΔΔ*Ct^ formula with *β*-actin as the internal control. Error bars represent the SD (*n* = 3) (^∗^*p* < 0.05, ^∗∗^*p* < 0.01, and ^∗∗∗^*p* < 0.001 vs. DPSCs; ^#^*p* < 0.05, ^##^*p* < 0.01, and ^###^*p* < 0.001 vs. PDLSCs; ^&^*p* < 0.05, ^&&^*p* < 0.01, and ^&&&^*p* < 0.001 vs. DFPCs; ^†^*p* < 0.05, ^††^*p* < 0.01, and ^†††^*p* < 0.001 vs. ABMMSCs).

For the chondrogenic capacity of DPSCs, PDLSC, DFPCs, and ABMMSCs, the MSCs were centrifuged in a 15 mL conical tube. After culturing with the cartilage differentiation medium for 21 days, the chondrocyte pellets were fixed and cut into paraffin sections. The sections were then stained with alcian blue, which indicated chondrogenic glycosides. All tested MSCs showed positive staining results (Figure 7(a)). We further evaluated the chondrogenic capacity of MSCs by comparing the relative mRNA expression of chondrogenic markers at days 0 and 21 of chondrogenic induction (Figure 7(b)). The results showed that all the chondrogenic markers were expressed highest in DPSCs.

## 4. Discussion

MSCs play a key role in the balance between health and disease [22]. By combining with different kinds of scaffold materials such as HAP and PLA, they provide a new treatment method for bone regeneration [23]. Considering their role in the tissues and organs, a lot of studies are devoted to exploring the basic biological mechanisms and potential clinical applications of MSCs [24–28]. The advantage of MSCs includes easy to harvest and does not tend to form tumors. When cultured in vitro under specific growth conditions, they also show the characteristics of self-renewal and differentiate into multiple cell types [29]. In addition, it has been proven that MSCs from different sources have significant immunomodulatory capabilities. In inflammatory diseases, MSCs produce a unique response by homing and integrating into damaged tissues [30]. These unique immunomodulatory properties make MSCs the main cell type in many clinical research fields [31]. More specifically, MSCs have shown great potential in the pathophysiology of bone injuries and diseases and can be used as a treatment option in future [32].

The evaluation of the best seed cells for tissue engineering includes many aspects. In addition to considering the characteristics of cells which contain proliferation and differentiating into specific lineages, it is also important to determine the source of the stem cells. Compared with the two somatic MSCs which are BMMSCs and ADSCs, dental-derived stem cells are becoming a promising cell source of regenerative medicine because the tissues can be obtained from medical waste such as wisdom teeth, which is simple and less moral controversy. At the same time, these organizations can be stored for use. For BMMSCs, additional invasive procedures may cause serious complications of the donor, such as infection. In addition, the lower cell mass can be obtained is a disadvantage encountered when isolating BMMSCs. Although ADSCs can be obtained by liposuction surgery or caesarean section, the progress is still invasive [33, 34]. In view of the above reasons, dental-derived stem cells perform better than somatic stem cell. This study is aimed at characterizing the biological characteristics and osteogenic differentiation potential of DPSCs, PDLSCs, DFPCs, and ABMMSCs and determining their application potential in bone regeneration. So far, such a direct comparison has not been reported.

In order to avoid individual differences, we isolated four types of cells from the same individual. Through the identification of cell surface markers and the ability of tri-lineage differentiation, the cells we isolated meet the standards of MSCs [5]. For cell therapy and tissue engineering applications which require a large number of cells, the proliferation ability of cells is very important. We evaluated the proliferation rate of four types of MSCs for consecutive 7 days. The results showed that all the MSCs reached the logarithmic growth phase at the third day and showed rapid growth thereafter. The proliferation rate of DFPCs was the fastest, DPSCs and PDLSCs were the second, and ABMMSCs were the slowest.

The antiapoptosis ability of stem cells is also an important aspect when considering therapeutic applications. It has been reported that about 99% of MSCs are lost in the first 24 hours after cell transplantation, and apoptosis is considered to be the main factor [35]. A lot of mechanisms may lead to apoptosis of transplanted cells, including nutrient deprivation and inflammatory environment [36]. We partially simulated the microenvironment of the transplantation site by using a serum-free medium and successfully induced MSC apoptosis. The results showed that ABMMSCs possessed the strongest antiapoptotic ability.

In addition, we studied the osteogenic differentiation capacity of DPSCs, PDLSCs, DFPCs, and ABMMSCs. Osteogenic differentiation ability was confirmed by ALP staining, ALP activity assay, and alizarin red staining. After osteogenic induction, ABMMSCs showed more ALP staining at day 7 and darker at day 14, followed by DPSCs and PDLSCs, and DFPCs possessed the lightest color. The ALP activity test results were consistent with the ALP staining. At day 21 of osteogenic induction, ABMMSCs showed stronger alizarin red staining, PDLSCs was second but slightly stronger than DPSCs, and DFPCs was the lightest. These results indicated that ABMMSCs produced more mineralized nodules in the process of osteogenic induction and were most sensitive to the differentiation of osteoblasts.

On the genetic level, we analyzed the expression of osteogenic markers (including ALP, RUNX2, COL-1, OCN, OPN, and OSX) to further prove osteogenic differentiation. The results showed that as the osteogenic induction time increased, the expression of osteogenic genes in ABMMSCs increased significantly and higher than DPSCs, PDLSCs, and DFPCs. At the same time, we analyzed the protein level at day 21 with or without osteogenic induction. At the late stage of osteogenic induction, the MSCs still show osteogenic potential. The expression of osteogenic proteins in ABMMSCs was significantly higher than that of DFPCs, followed by PDLSCs and DPSCs. In summary, our results showed that compared with the abovementioned four types of dental-derived stem cells, ABMMSCs possessed the strongest osteogenic capacity, PDLSCs were slightly weaker, DPSCs were second, and they were all better than DFPCs. Considering the availability of cell sources, proliferation, apoptosis, and osteogenic ability, ABMMSCs and PDLSCs are considered to be potential substitutes for BMMSCs in bone tissue engineering.

## 5. Conclusions

In summary, our results indicated that DPSCs, PDLSCs, DFPCs, and ABMMSCs from the same individual possessed different biological characteristics. ABMMSCs and PDLSCs showed a higher osteogenic potential, which would make them the optimal stem cells for bone tissue engineering. However, we have only compared the biological characteristics and osteogenic potential of these stem cells in vitro. More research needs to be done in vivo to characterize the optimal stem cells source for bone regeneration.

## Figures and Tables

**Table 1:** All primers used for qRT-PCR.

Genes	Forward (5′-3′)	Reverse (5′-3′)
RUNX2	TGGTTACTGTCATGGCGGGTA	TCTCAGATCGTTGAACCTTGCTA
ALP	GTGAACCGCAACTGGTACTC	GAGCTGCGTAGCGATGTCC
OCN	GAAGTTTCGCAGACCTGACAT	GTATGCACCATTCAACTCCTCG
OPN	GAAGTTTCGCAGACCTGACAT	GTATGCACCATTCAACTCCTCG
COL-1	TGCTCGTGGAAATGATGGTG	CCTCGCTTTCCTTCCTCTCC
OSX	CCTCTGCGGGACTCAACAAC	AGCCCATTAGTGCTTGTAAAGG
APN	GGCTTTCCGGGAATCCAAGG	TGGGGATAGTAACGTAAGTCTCC
C/EBP*α*	GTGGACAAGAACAGCAACGA	GGTCATTGTCACTGGTCAGC
FABP4	ACTGGGCCAGGAATTTGACG	CTCGTGGAAGTGACGCCTT
COL-2	TTTCCCAGGTCAAGATGGTC	CTTCAGCACCTGTCTCACCA
SOX9	AGCGAACGCACATCAAGAC	CTGTAGGCGATCTGTTGGGG
ACAN	TGAGGAGGGCTGGAACAAGTACC	GGAGGTGGTAATTGCAGGGAACA
*β*-Actin	TGGCACCCAGCACAATGAA	CTAAGTCATAGTCCGCCTAGAAGA

## Data Availability

The authors declare that all the data supporting the findings in this study are available from the corresponding author through email on reasonable request.

## Abbreviations

MSCs: Mesenchymal stem cells
DPSCs: Dental pulp stem cells
PDLSCs: Periodontal ligament stem cells
DFPCs: Dental follicle progenitor cells
ABMMSCs: Alveolar bone marrow mesenchymal stem cells
PFA: Paraformaldehyde
qRT-PCR: RNA extraction and quantitative real-time polymerase chain reaction
ALP: Alkaline phosphatase
COL-1: Collagen type I
COL-2: Collagen type II
ACAN: Aggrecan
RUNX2: Runt-related transcription factor 2
OSX: Osterix
OCN: Osteocalcin
OPN: Osteopontin
APN: Adiponectin
FABP4: Fatty acid-binding protein 4
CPC: Cetylpyridine chloride.
